# Longitudinal Changes of Cardiorespiratory Fitness Performance in High School: Association with Individual and School-Based Variables

**DOI:** 10.3390/children9121884

**Published:** 2022-12-01

**Authors:** Xihe Zhu, Justin A. Haegele, Jinting Shao, Summer Davis

**Affiliations:** 1 Department of Human Movement Sciences, Old Dominion University, Norfolk, VA 23529, USA; 2 Department of Physical Education, Zhejiang University of Finance & Economics, Hangzhou 310012, China

**Keywords:** adolescent youth, academic achievement, aerobic capacity, physical education, sex, school health

## Abstract

This study aimed to model adolescents’ cardiorespiratory fitness performance change trajectories longitudinally across high school years and its relation to school- and individual/student-level factors. We employed hierarchical linear modeling to examine longitudinal cardiorespiratory fitness performance changes, as measured by the progressive aerobic capacity endurance run (PACER), over the years, between sexes, and in association with the school-level variables. Participants were 76,227 adolescents from 80 high schools in the mid-Atlantic region of the United States. School-level academic performance (SAP), the percent of students eligible for free and reduced-price meals (FARM), and physical education student-faculty ratio were obtained with permission from the school districts. The number of laps completed in PACER test improved throughout the first three years of high school, however, proportions of those within the healthy fitness zone (HFZ) decreased overall from 9th to 11th grade. Furthermore, the number of laps completed by adolescents appeared to have plateaued at 11th grade, with a significant decline during the final year of high school. Sex-based discrepancies in performance in meeting HFZ were evident, where girls significantly outperformed boys during 9th and 10th grades, and boys significantly outperformed girls during 12th grade. Additionally, SAP and FARM were positively and negatively, respectively, significantly associated with PACER performance at the school level. The odds ratio of adolescents performing in the HFZ declined significantly over the years, even though the number of PACER laps improved in the first three years. Concerted efforts should be targeted at improving cardiorespiratory fitness in high school due to its positive relationship to academic achievement in schools, and negative association with cardiovascular disease, metabolic syndrome, obesity, and all-cause mortality in adulthood.

## 1. Introduction

Cardiorespiratory fitness (CF), a marker of cardiovascular health that provides a measure of the ability of the circulatory and respiratory systems to supply oxygen to skeletal muscles during sustained physical activity [1], has been identified as an important health-related component of physical fitness that may be associated with health concerns throughout the lifespan [2,3,4,5,6]. For example, among high school-aged adolescents, favorable CF is associated with lower cardiometabolic risk, overall adiposity, and risk of metabolic syndrome development [7,8,9]. Furthermore, research has shown that CF during the high school years may predict cardiovascular disease, obesity, and all-cause mortality in adulthood [10]. In addition to health-related associations, evidence supports CF as being positively associated with brain vascularization and enhanced cognition among high school-aged adolescents, which may influence improved academic performance [11,12,13,14]. Despite the explicated benefits of monitoring and maintaining CF [6,7,8,9,10,11,12,13], recent evidence has shown a secular decline in CF internationally among adolescents and adults, particularly among males, over the past 50 years [2,15]. This decline is suggestive of a potential decrease in population health [15], as health-related benefits associated with proper CF are less likely to be garnered [6,7,8,9,10].

It is clear that there are existing studies that have examined CF of the youth population [5,6], the findings may be limited by a number of factors. Notably, while longitudinal evidence is available that examines the effects of childhood and adolescent CF on health-related risk factors in adulthood [10], limited longitudinal evidence is available that examines CF performance changes throughout students’ academic careers. Most studies focusing on high school-aged individuals have utilized cross-sectional designs, or have examined secular trends, and have not tracked CF changes throughout the high school years. One longitudinal study, which examined cardiorespiratory changes from middle to high school among adolescent girls, identified CF decreased from 9th to 12th grade [16]. A second study, which focused specifically on middle school-aged students, showed CF improvement, but at a slower rate than that of the criteria for the Healthy Fitness Zone (HFZ) of their age group and identified different growth curves for boys and girls [17]. Understanding trends in CF during the high school years is of prime importance, as this is a critical time in the development of behaviors and attributes that will carry over into adulthood. Hence, the primary purpose of this study was to examine the longitudinal changes in CF among high school-aged students.

Investigation into how school-level factors influence cardiorespiratory performance among high school students is also warranted [16,18]. For example, the school environment, including class sizes, the availability of sports fields or gymnasia, days of physical education per week, and community location (i.e., urban, suburban, rural), have been associated with physical fitness scores, including CF performance, among high school students [18,19]. Theoretically, the amount teaching staff and resources that school have would influence how well students perform in the fitness and academically as well. However, other school-level factors, such as student-faculty ratio and poverty level that are critical factors for school-level academic performance [20,21] have only been sporadically examined in their association with CF and CF knowledge among high school-aged students [22,23]. Furthermore, the dearth of longitudinal large-scale studies examining the association with school-level factors limits our understanding of changes in CF that occur during high school years. Hence, another aim of the study was to explore the CF performance changes and their association with student demographic as well as school-based socioeconomic variables.

## 2. Methods

### 2.1. Study Design and Procedures

This observational study utilized existing datasets that contained multilevel, longitudinal CF data, which were accumulated over a four-year period when adolescents in high school were tested annually as they moved from ninth to twelfth grade. Data from the students were collected by certified health and physical education teachers, who entered the data into an online system named Welnet^®^ (Focused Fitness LLC, Spokane Valley, WA, USA; https://reports.focusedfitness.org/WELNET/login.php, accessed on 30 August 2022). An acceptable level of consistency in validity and reliability of expert and teacher administered 20 m Progressive Aerobic Cardiovascular Endurance Run (PACER) tests has been documented [24], supporting the utilization of teacher collected data. In addition to PACER scores, the online system also recorded data on participants’ demographic information such as age, grade level, sex, school, and test dates that were synced with the district data. The student-level data were not directly collected by the researchers, but researchers sought and received permission to access and analyze the de-identified student data from the school districts. School-level factors, including the percent of students eligible for FARM, physical education student-faculty ratio (S/F-PE), and SAP were included in this study as well. Data related to school-level factors were gathered via school academic reports on the webpages of the school districts.

Schools were included in this analysis if (a) fitness education units had been taught in their physical education curriculum for the past four years and (b) fitness testing such as CF was required and assessed annually [24]. Student data were included in this analysis if they completed two or more years for their CF tests [25] over the four-year period. Because the school districts mandated physical fitness assessments for all public-school students during ninth and tenth grade, the sample represented each districts’ population of student very well, accounting for about 60.7% of the overall enrollment in these 80 schools. However, physical education, and subsequently physical fitness testing, was not mandatory in most districts during eleventh and twelfth grade, and therefore participation decreased dramatically during these years, as shown in Table 1. The study was review and approved by the Old Dominion University Darden College of Education and Professional Studies Human Subjects Review Committee (#872750), under exempt category for using de-identified common educational assessment data.

### 2.2. Participants

There were 76,227 adolescents (47.7% female) from 80 high schools from the Mid-Atlantic region of the United States who participated the study. They averaged 15.08 ± 0.73 years old (range: 13–19) and were enrolled at the 9th- through 12th-grade levels. The racial/ethnic percentage at the school level was 10.74% Asian/Asian American, 19.64% Black/African American, 11.36% Latino/Hispanic, 51.39% White/Caucasian, and 6.84% Others. The schools were socioeconomically diverse, with FARM rate ranging from 1.79% to 72.61% at the school level.

### 2.3. Variables and Measures

To model longitudinal changes, we included individual/student- and school-level variables. Student-level variables were grade (testing year), sex, and 20 m PACER score, which was tested annually. The PACER test is a criterion-referenced field evaluation of CF that is recommended for ease of use and convenience accommodation needs [24,25]. The PACER is a multistage aerobic capacity assessment that consists of warm-up, then, starts at a slow pace but gradually progresses toward a faster pace. Participants start from one side then run across a 20 m area passing another end line before a beep sounds, then continue to run between end lines until they could not get to the opposite side before the beep, twice. The PACER test performance level was evaluated by scoring the number of laps that the students successfully completed. The number of laps is then used to determine if participants performed within an HFZ [25], which includes age-and sex-specific criteria for CF, or not.

We collected school-level variables including FARM, SAP, and S/F-PE from the websites of the school districts and/or state department of education. S/F-PE was calculated through dividing the school enrollment by the number of fulltime health and physical educators employed. For SAP, school-level passing rate (%) in statewide assessments for reading, mathematics, science, and social science for the previous three years were collected. Given that passing rates were highly correlated at the school level, with the correlation coefficient *r* ranging from 0.57 to 0.82 among the 4 tested areas, we computed an aggregated average passing rate of those subject areas for each school. Specifically, for each school, SAP was computed as $\sum_{1}^{i}\sum_{1}^{j}Subject_{ij}/ij$, where the pass rate of ith subject (i = 4) passing rate for jth year (j = 4) for the past 4 years was summed and then averaged.

### 2.4. Statistical Analysis

We used hierarchical linear modeling (HLM) to model longitudinal CF performance change over the years, between sexes, and in association with the school-level variables [26]. Since the PACER was tested at the student level multiple times, a three-level HLM was used to model PACER performance change across years in relation to individual/student- and school-level factors. Specifically, the school-level variables were grand mean centered using z scores. Model and statistical assumptions were based on recommended practices, and model specifications were screened prior to model testing [26]. We checked Mahalanobis distance and P-P plot for normality, scatterplot for linearity, and residual Q-Q plot for homoscedasticity. Then, we began with fitting a full unconditional model, which provided information for adding further parameters for Level 1, Level 2, and Level 3. Full information maximum likelihood estimation was used to compute the variance covariance components. Eventually, Level 1, with an individual PACER performance modeled at time t of participant i in school j, was specified:

Y_tij_ = π_0ij_ + π_1ij_ (Grade-9)_tij_ + π_2ij_ (Grade-9)_tij_^2^+ e_tij_ (1)

where Y_tij_ is a logit variable, defined as ln(p/(1−p)), where p is the probability of PACER performance in HFZ at time t for participant i in school j. Level one model is centered on 9th grade, where π_0ij_ is the estimated initial logit for child i in school j at grade 9, π_1ij_, π_2ij_ are the first-order, and second-order growth rate for participant ij during the academic year; and e_tij_ is the Level 1 random error. At Level 2, we specified the model to examine the sex difference:

π_tij_ = β_tij_ + β_tij_(Sex)_ij_ + r_tij_ (2)

To model the sex differences, β_tij_ values represented the mean PACER performance and the first-order, and second-order growth rate differences within school j. Specifically, we coded 0 for girls, and 1 for boys based on sex data. The Level 2 random error included r_tij_. We specified the Level 3 model to examine the association between school-level variables such as FARM, SAP and PACER performance:

*β*_00j_ = *γ*_000_ + *γ*_001_(FARM)_j_ + *γ*_002_(S/F-PE)_j_ + *γ*_003_(SAP)_j_ + u_00j_ (3)

At level 3, the *γ*_00j_ were the average school-level performance and coefficients associated with a variable, holding other school-level variables constant. The Level 3 random error was u_00j_. We hypothesized in the model that school-level variables FARM, S/F-PE, and SAP were associated with PACER performance, and that there were sex differences in growth rates of the performances at individual level. We conducted the data analyses using HLM version 6.08 (Scientific Software International; Skokie, IL, USA), and kept α = 0.05.

## 3. Results

Participants’ PACER performance (# of lap completed, Table 1) on average increased from grade 9 to grade 11, but it declined at grade 12. As shown in Table 1, the number of students completing the PACER decreased substantially at grade 11 when physical education (along with physical fitness assessment) was no longer required. When modeling sex differences and school-level variables, we noticed that SAP was highly correlated with school FARM with r = −0.80. As such, in the final model testing, we added SAP, then FARM independently. The model testing results are shown in Table 2.

There was a sex difference and significant school-level variable impact on whether adolescents performed in the HFZ. In general, girls were more likely to be in the HFZ than boys (OR = 1.74). Controlling for the school-level variables, boys and girls had significantly different second-order curves for being in the HFZ while they shared a concaving downward pattern. SAP was positively associated with adolescent PACER performance (*γ* = 0.26). Adolescents in schools with higher SAP were more likely to be in the HFZ (OR = 1.29). When FARM was added to the model, as shown in the bottom panel in Table 2, FARM was negatively associated with PACER performance (*γ* = −0.59), while SAP was no longer statistically significant (*p* = 0.09) suggesting that they were confounding factors at school level. Participants in schools with higher FARM were less likely to be in HFZ (OR = 0.55) than those in schools with lower FARM. While the new model fits better than the one with SAP alone (χ^2^ = 4.90, Δdf = 1), the amount of variance explained was only marginally improved to 0.51 (Δ*r*^2^ = 0.01).

**Figure 1:**
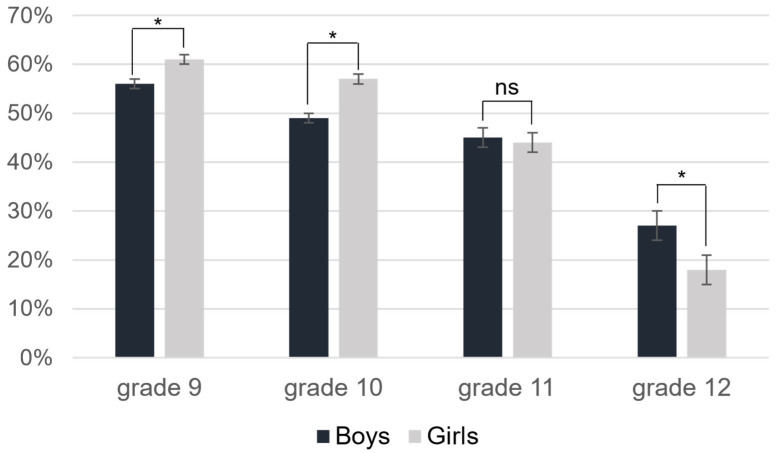
Four-year percentage changes of cardiorespiratory fitness performance in healthy fitness zone, * *p* < 0.05.

The estimated percentage and odds ratio for adolescents in the HFZ as they advanced from grade 9 to grade 12 are presented in Table 3 and Figure 1. In general, adolescents were significantly more likely to be in the HFZ in grades 9, 10, and 11 than they were in grade 12, for both boys and girls. Among different grade levels, girls had a higher proportion in the HFZ than boys for grades 9 and 10, while boys had a higher proportion than girls in grade 12. As displayed in Figure 1, there was no sex difference for grade 11. Finally, while the number of laps adolescents completed increased from grade 9 to 11 in PACER (Table 1), the percentage and odds ratio of those in HFZ declined (Figure 1, Table 3). In particular, adolescents’ performance (# of laps completed) had a significant decline in grade 12 than those of grade 11 on average.

## 4. Discussion

In this study, we reported CF changes among adolescents in high school, through analyzing a longitudinal, multilevel dataset. In general, participants in this study were not highly likely to perform in the HFZ. In fact, at the peak of the participants’ performance, just 56% of boys and 61% of girls performed in the HFZ. These findings may support recent evidence showing a decline in CF internationally [2], which is reflective of a decline in population health in general [15]. Consistent with the extant literature [2,15,17,23,27], sex-based discrepancies in performance in the HFZ were found in this study. With girls being 1.74 times more likely to be in the HFZ than boys overall, sex-based differences were particularly pronounced during 9th and 10th grade. As participants progressed through school, however, sex-based differences were absent during 11th grade, and inverted during 12th grade, where boys were more likely to meet HFZ standards than girls. It should be noted that biological changes, such as body size and maturation, that occur throughout the high school years may contribute to sex-based differences between boys’ and girls’ performance [28]. Furthermore, social factors, such as pressure to maintain and display what conventional conceptions consider an ideal female body (e.g., slenderness, limited muscularity), may influence adolescent girls’ motivation to participate and how they perform in cardiorespiratory testing [22]. Thus, as noted by Armstrong and Welsman, analyzing CF by sex and age alone may be limited, as it ignores other factors such as body size, motivation, and maturation independent of age that may influence performance [28].

The results of this study revealed an alarming decline in cardiorespiratory performance in the HFZ among the adolescent participants. While there were increases in completed number of laps in the PACER across participants over the first three years of the analysis, they were less likely to remain in the HFZ over time. Meaning, although participants were able to complete more laps, they did not improve at a rate that was considered healthy for their age groups. Of concern, by age 16 (11th grade) most participants fell below the established HFZ, the lower end cutoff score. Therefore, the favorable health effects of HFZ CF levels [6,7,8,9] may have been limited for these participants in this study. This decline is consistent with previous studies [16,17], which have identified that adolescents tend to experience a decline in CF as they progress through the middle and high school years. Of additional concern, performance plateaued at grade 11, and a significant decline in those performing in the HFZ occurred in grade 12. These concerns are heightened when considering the participants in this study were those enrolled in volitional physical education classes or those needing the physical education credit for high school graduation, a setting theorized to help enhance fitness and cardiorespiratory endurance [29], during 11th and 12th grade. However, the findings of this study support prior assertions that engagement in physical education classes may do little to affect students’ CF [29,30]. Thus, by the time participants completed the 12th grade, few would experience the health-related [6,7,8,9] and academic performance benefits associated with favorable CF [11,12,13].

The second goal of our study was to explore the potential association between individual/student- and school-level variables and CF performance among high school-aged students. In addition to sex differences at the student level, school-level variables were associated with performance in the HFZ. In this study, SAP was positively associated with likelihood of student performing in the HFZ, where those enrolled in schools with higher SAP were more likely to perform in the HFZ (OR = 1.29). This positive association contributes to the growing evidence of the positive aspect of higher CF, and other health-related fitness components, on academic performance, small sometimes at individual level, but significant at school level [17,31,32]. Conversely, FARM was negatively associated with adolescent likelihood in the HFZ, which contributes to issues regarding lower health-related fitness performance in poorer schools that may have less resources and larger class sizes [17,19]. It should be noted that SAP and FARM were confounding variables at the school level in this study, suggesting that highly performing schools with high rates of FARM may have counteracted the negative effects typically associated with poorer schools.

The research was able to address some known limitations of the existing studies. Notably, we had the opportunity to examine a large dataset that included longitudinal data and multilevel variables and examined the association with school-level variables on adolescent fitness performance (in the HFZ). The availability of data across four years of high school allowed the researchers to study likelihood of HFZ changes across those years, which adds a longitudinal dimension that researchers desired [33]. Nevertheless, this study also has its limitations. For example, Armstrong and Welsman have recently challenged the value of utilizing the PACER as a suitable measure, citing that it (mis)represents and therefore is (mis)interpreted youth CF [34]. Conflictingly, however, our views of the PACER align with those of Tomkinson and colleagues who note the PACER as a “simple, single measure that assesses the integrated responses of the physiological systems’ ability to perform progressive aerobic exercise” (p. 1) which has strong utility and scalability for population health surveillance [35]. Furthermore, the drastic decrease in sample size in 11th and 12th grade, while in general alignment with a national report [36], should be noted as a limitation as only limited participants completed CF assessment in these two years. In addition, the results of this study reported changes in CF performance and adolescent likelihood in HFZ under natural physical education in public school settings, with no control group or experimentation, or controlling for body size and maturation independent of age. Thus, the findings should be interpreted with a caution for not overgeneralizing them to cause-effect relationships.

In conclusion, this study indicates that although PACER performance (# of laps completed) improved throughout the first three years of high school, the likelihood of those performing within or above the HFZ decreased overall from 9th to 11th grade. Furthermore, the number of laps completed by adolescents appeared to plateau among participants at 11th grade, with a steep decline in performance during the final year of high school. Additionally, SAP and FARM were positively and negatively associated with performance in the HFZ, respectively. These findings have practical implications for school districts and scholars examining CF among high school-aged students.

## 5. Conclusions

The findings from this study illustrate declining trend of high school student meeting HFZ, and the need to help high-school adolescents to maintain CF which has shown to be positively correlated to multiple physical and mental health and academic performance. The findings also show factors at the school and individual level that are associated with the probability of the students achieving HFZ. These findings are intertwined and have several implications that would help improve CF and school health in general.

Considering the declining rate of students achieving HFZ, it is important that school-based programs including those before, after school, or during school such as physical education and recess have built physical activity elements that are purposefully designed to improve student physical fitness. Promoting and implementing scalable evidence-based programs would not only benefit adolescents on their physical fitness, but also benefit school and the society, particularly during late high school years as these students are entering the labor force and/or higher education as young adults.

At the school level, it is important for school administrators to recognize the importance of student physical fitness such as CF on academic achievement which was confounding variable with FARM. The lack of physical educators as indicated by the high student/faculty ratio and discontinuance of offering required physical education during higher grades in our research context further limited students’ opportunity to engage in activities to improve or maintain their physical fitness. Extending more physical education or physical activity programs to students in higher grades (e.g., 12th grade) would help these students as move into early adulthood. For schools with higher FARM, these physical activity programs which would help improve physical fitness and academic performance, would be particularly recommended.

## Figures and Tables

**Table 1:** Participation information for PACER testing age and grade.

Testing Year	Age (M ± SD)	Participant (N)	PACER (# Laps, M ± SD)
Grade 9	14.61 ± 0.69	76,227	41.44 ± 21.10
Grade 10	15.41 ± 0.70	73,602	43.74 ± 21.71
Grade 11 *	16.38 ± 0.92	5495	45.81 ± 23.64
Grade 12 *	17.63 ± 0.70	1372	40.67 ± 22.35

Note: * Physical education was elective for these grade levels. PACER = 20 m Progressive Aerobic Cardiovascular Endurance Run.

**Table 2:** Youth PACER performance changes through ninth to twelfth grades.

Fixed Effect with SAP	Coefficient	*se*	*t* Ratio	*df*	OR (95% CI)	*p* Value
Model for performance, **π**_0ij_						
Predicting *β*_00j_						
Intercept, *γ*_000_	0.55	0.09	6.21	77	1.74 (1.46, 2.07)	<0.001
S/F-PE, *γ*_001_	−0.06	0.07	−1.03	77	0.94 (0.85, 1.05)	0.306
SAP, *γ*_002_	0.26	0.07	3.73	77	1.29 (1.13, 1.49)	0.001
Predicting *β*_01j_						
Intercept sex, *γ*_010_	−0.23	0.05	−4.11	81,557	0.79 (0.71, 0.88)	<0.001
Model for growth rate (1st order), **π**_1ij_						
Predicting *β*_10j_						
Intercept, *γ*_100_	−0.10	0.08	−1.24	156,688	0.91 (0.77, 1.06)	0.215
Predicting *β*_11j_						
Intercept sex, *γ*_110_	−0.18	0.07	−2.40	156,688	0.83 (0.72, 0.97)	0.016
Model for growth rate (2nd order), **π**_2ij_						
Predicting *β*_20j_						
Intercept, *γ*_200_	−0.09	0.04	−2.37	156,688	0.91 (0.85, 0.98)	0.018
Predicting *β*_21j_						
Intercept sex, *γ*_210_	0.10	0.04	2.51	156,688	1.10 (1.02, 1.19)	0.012
**Fixed Effect with FARM and SAP**	**Coefficient**	***se***	***t*** **Ratio**	***df***	**OR (95% CI)**	***p*** **Value**
Model for performance, **π**_0ij_						
Predicting *β*_00j_						
Intercept, *γ*_000_	0.57	0.08	7.08	76	1.76 (1.51, 2.07)	<0.001
FARM, *γ*_001_	−0.59	0.13	−4.30	76	0.55 (0.42, 0.73)	<0.001
S/F-PE, *γ*_002_	−0.06	0.05	−1.11	76	0.94 (0.85, 1.05)	0.270
SAP, *γ*_003_	−0.24	0.14	−1.71	76	0.78 (0.59, 1.04)	0.091
Predicting *β*_01j_						
Intercept sex, *γ*_010_	−0.24	0.06	−4.15	81,557	0.79 (0.71, 0.88)	<0.001
Model for growth rate (1st order), **π**_1ij_						
Predicting *β*_10j_						
Intercept, *γ*_100_	−0.10	0.08	−1.23	156,688	0.91 (0.77, 1.06)	0.217
Predicting *β*_11j_						
Intercept sex, *γ*_110_	−0.18	0.07	−2.36	137,260	0.84 (0.72, 0.97)	0.019
Model for growth rate (2nd order), **π**_2ij_						
Predicting *β*_20j_						
Intercept, *γ*_200_	−0.09	0.04	−2.41	137,260	0.91 (0.85, 0.98)	0.016
Predicting *β*_21j_						
Intercept sex, *γ*_210_	0.10	0.04	2.53	137,260	1.11 (1.02, 1.20)	0.012

Note: CI = Confidence Interval; OR = Odds ratio; S/F-PE = Student Faculty Ratio for Physical Education; SAP = School Academic Performance (% of student passing state test).

**Table 3:** Estimated proportion and odds ratio of youth in healthy fitness zone (HFZ).

Sex	Year	% in HFZ (95% CI)	Odds Ratio in HFZ (95% CI)
	Grade 9	0.56 (0.55, 0.56)	2.32 (1.77, 3.03) **
Boys	Grade 10	0.49 (0.48, 0.49)	2.14 (1.64, 2.79) **
	Grade 11 ^†^	0.45 (0.43, 0.47)	1.64 (1.23, 2.18) *
	Grade 12 ^†^	0.27 (0.24, 0.30)	1.00 (referent)
	Grade 9	0.61 (0.61, 0.62)	3.35 (2.88, 3.91) **
Girls	Grade 10	0.57 (0.56, 0.57)	2.56 (2.20, 2.98) **
	Grade 11 ^†^	0.44 (0.42, 0.46)	2.21 (1.87, 2.61) *
	Grade 12 ^†^	0.18 (0.15, 0.21)	1.00 (referent)

Note: ^†^ Physical education was elective for these grade levels; * *p* < 0.05; ** *p* < 0.01; CI = confidence interval.

## Data Availability

The data are not publicly available due to lack of permission from the participating school districts and the research ethics regulations at the corresponding author’s institution.
